# The Inhibitory Effect of Salmon Calcitonin on Tri-Iodothyronine Induction of Early Hypertrophy in Articular Cartilage

**DOI:** 10.1371/journal.pone.0040081

**Published:** 2012-06-29

**Authors:** Pingping Chen-An, Kim Vietz Andreassen, Kim Henriksen, Yadong Li, Morten Asser Karsdal, Anne-Christine Bay-Jensen

**Affiliations:** 1 Cartilage Biology and Biomarkers, Nordic Bioscience A/S, Herlev, Denmark; 2 Bone Biology and Pharmacology, Nordic Bioscience A/S, Herlev, Denmark; 3 Orthopedic Surgery Unit, Beijing Friendship Hospital, Beijing, People’s Republic of China; University of Texas MD Anderson Cancer Center, United States of America

## Abstract

**Objective:**

Salmon calcitonin has chondroprotective effect both *in vitro* and *in vivo,* and is therefore being tested as a candidate drug for cartilage degenerative diseases. Recent studies have indicated that different chondrocyte phenotypes may express the calcitonin receptor (CTR) differentially. We tested for the presence of the CTR in chondrocytes from tri-iodothyronin (T3)-induced bovine articular cartilage explants. Moreover, investigated the effects of human and salmon calcitonin on the explants.

**Methods:**

Early chondrocyte hypertrophy was induced in bovine articular cartilage explants by stimulation over four days with 20 ng/mL T3. The degree of hypertrophy was investigated by molecular markers of hypertrophy (ALP, IHH, COLX and MMP13), by biochemical markers of cartilage turnover (C2M, P2NP and AGNxII) and histology. The expression of the CTR was detected by qPCR and immunohistochemistry. T3-induced explants were treated with salmon or human calcitonin. Calcitonin down-stream signaling was measured by levels of cAMP, and by the molecular markers.

**Results:**

Compared with untreated control explants, T3 induction increased expression of the hypertrophic markers (p<0.05), of cartilage turnover (p<0.05), and of CTR (p<0.01). Salmon, but not human, calcitonin induced cAMP release (p<0.001). Salmon calcitonin also inhibited expression of markers of hypertrophy and cartilage turnover (p<0.05).

**Conclusions:**

T3 induced early hypertrophy of chondrocytes, which showed an elevated expression of the CTR and was thus a target for salmon calcitonin. Molecular marker levels indicated salmon, but not human, calcitonin protected the cartilage from hypertrophy. These results confirm that salmon calcitonin is able to modulate the CTR and thus have chondroprotective effects.

## Introduction

The pathogenesis of cartilage loss in joint degenerative diseases is not fully understood, but experimental and clinical studies have shown that abnormal subchondral bone turnover and cartilage calcification affects the integrity of the articular cartilage structure [1]–[3]. In particular, hypertrophic differentiation of chondrocytes, leading to cartilage calcification, is a key event of the deep layer of the cartilage in early joint deterioration [4]–[5]. Because of the strong inter-relationship between cartilage and subchondral bone, an ideal agent for joint degenerative diseases would have positive effects on both remodeling subchondral bone and the prevention or regeneration of articular cartilage. However, few drugs are available to effectively prevent or treat cartilage degradation [6].

Calcitonin is a part of the calcitonin family consisting of calcitonin (CT), α-calcitonin gene-related peptide (α-CGRP), βCGRP, adrenomodullin (AM), and amylin [7]. In mammals, these peptides signal through two closely related type II GPCRs (Calcitonin Receptor and Calcitonin Receptor-like Receptor) and three unique receptor activity-modifying proteins (RAMPs) [8]. The combination of receptor and RAMP determines the sensitivity of the receptor towards stimulation with the individual ligands, i.e. the CTR with no RAMP binds human CT strongly, while the CTR in the presence of RAMP1 is a receptor for AMY [9]. Human CT, salmon CT, and amylin share receptors [10]; however, what separates salmon CT from human CT is an intrinsic ability to bind to and activate the CTR in the presence of RAMPs, most notably in the confirmation normally activated potently by Amylin [11]–[12].

The literature contains some controversies on calcitonin receptor (CTR) expression in chondrocytes [13]–[15]. This may, for the major part, be due to the different in vitro and ex vivo system applied; Lin et al. [15] used primary cultured chondrocytes, whereas Sondergaard et al. [14] used freshly isolated OA chondrocytes. Both systems have its limitations. Segovia-Silvestre et al. [13] showed that primary chondrocytes expressed the CTR, both transcriptionally and translationally. Furthermore, it was shown that the CTR could be found in OA cartilage tissue sections *in situ*.

Calcitonin is widely used as an anti-resorptive agent for the treatment of osteoporosis [16]–[17]. The potential use of calcitonin in the treatment of joint degenerative diseases is being investigated both pre-clinically and clinically. It has been found that calcitonin has a chondroprotective effect *in vitro* [18]; [19] as it inhibits matrix metalloproteinase (MMP) expression in articular cartilage, and prevents net loss of collagen, hyaluronan and proteoglycan aggregated from cartilage. Calcitonin has also been shown to promote growth of endochondral cartilage and maturation of growth plate cartilage [20]–[21]. Treatment with calcitonin has been shown to protect against bone loss and reduce osteoarthritis (OA) lesions in cartilage *in vivo*; suggesting that prevention of subchondral bone loss contributes to protection of cartilage integrity [22]–[24]. The effect of calcitonin on hypertrophic articular cartilage is yet to be investigated.

Many of the signaling pathways identified in endochondral ossification are also believed to be important in OA progression through regulation of chondrocyte hypertrophy [25]–[28]. Bone morphogenic protein (BMP) signaling, fibroblast growth factor (FGF) signaling and systemic factors like thyroid hormone together with a negative feedback loop like IHH/PTHrP are the main regulatory pathways of growth plate chondrocyte proliferation and hypertrophy in endochondral ossification [29]–[30]. BMP2 is suggested to be a strong mediator of chondrocyte hypertrophy, which is characterized by increased type X collagen (COLX) and alkaline phosphatase (ALP) expression, as well as up-regulation of Indian hedgehog expression (IHH) [31]–[32]. In contrast, basic FGF (bFGF) has been shown to inhibit hypertrophy by down-regulating IHH expression secreted by pre-hypertrophic chondrocytes [29]; [33]. Numerous evidence has confirmed the effect of tri-iodothyronine (T3) on terminal differentiation of chondrocytes [29]; [34]–[36]. These factors affect chondrocyte behavior in together with locally secreted IHH and PTHrP. In addition, ascorbic acid combined with β-glycerophosphate show a potency to induce chondrocyte hypertrophy [37]. Nevertheless, the mechanisms of these signaling pathways or factors modulating chondrocyte hypertrophy in the pathogenesis of OA still need to be elucidated.

We recently presented that T3 could induce chondrocyte hypertrophy in a full-depth cartilage explants model more effectively than bone morphogenic protein 2 (BMP2) and basic fibroblast growth factor (bFGF). We found that treatment for 4 days with T3 induced a pre-hypertrophic and proliferative phenotype in articular cartilage explants, whereas treatment for 8 or more induced a calcificatious and apoptotic phenotype. The characterization was performed by monitoring the expression pattern of common hypertrophic [31]–[32] and degenerative biomarkers [38]–[40]. In the present study we tested the hypothesis that a hypertrophic cartilage phenotype would express more CTR and thus respond to calcitonin.

## Materials and Methods

### Reagents

Most of the reagents were obtained from Sigma-Aldrich (Denmark), including: phosphate buffered saline (PBS), T3, collagenase and alkaline phosphatase assay reagents. Pronase was bought from Roche (UK). Tumor necrosis factor α (TNF-α) was obtained from R&D Systems (UK). Dulbecco’s Modified Eagle Medium, Nutrient Mixture F-12 (DMEM:F12) was purchased from Invitrogen (Denmark). Antibiotics were obtained from Life Technologies (Denmark).

### Full-depth Cartilage Explants Culture

Bovine full-depth cartilage (FDC) explants were harvested from the proximal femoral condyle of the hind leg of cows aged ≤1.5-years, retrieved from e local butchery 1–2 days after killing (Harald Hansen, Slangerup, Denmark). A skin punch biopsy (Miltex, Germany) 3 mm in diameter was used to take full depth cartilage plugs (i.e. explants). The isolated explants weighing 20∼30 mg were washed three times in sterile PBS and cultured 24 hours with DMEM:F12 with antibiotic, without serum, in 96-well plates at 37°C in 5% CO_2_. To induce chondrocyte hypertrophy, 20 ng/mL T3 was added to the explants and incubated for 4 days. Two calcitonin stimulation experiments were performed: i) Co-stimulation with 1 nM or 10 nM salmon calcitonin (Bachem, Germany) during the entire 4-day culture period. Explants cultured in DMEM:F12 with antibiotics with T3 were used as controls (W/O). At the end of experiment, the plugs were used for either histology or RNA isolation. ii) Induction of the CTR on day 4 or 8 (by two-hour stimulation with either 10 nM human or salmon calcitonin. For all experimenters the culture medium was changed three times a week and the collected supernatants were stored at -20°C for further analysis.

### Cell Viability

Alamarblue® (Invitrogen, US) was used as a non-toxic reagent for measuring cell viability and metabolic activity after the preincubation period. After removal of the medium, a 10% Alamarblue reagent in 10 mM PBS was added as 200 µl/well in a 96-well plate and incubated with explants for two hours at 37°C in 5% CO2. Then 160 µl was transferred to black microtiter plates and the fluorescence was read at 540 nm excitation 590 nm emission wavelength. The readout was normalized to the size of each explant (in mg). The remaining 40 µl was discarded and the explants were washed three times in culture medium.

### Histological Analysis

#### Toluidine Blue staining

Cultured bovine FDC explants were isolated at each time point and snap-frozen in liquid nitrogen. Frozen sections (5 µm) were mounted on superfrost^+^ slides, fixed in ice-cold acetone, and stained with Toluidine Blue. Digital histographs were taken with an Olympus BX60 microscope and an Olympus C5050-zoom camera. Perimeter measurement was done from 20X pictures using a free-ware program for measuring pixels.

#### Immunohistochemical detection of the CTR

The cultured bovine FDC explants were fixed in 4% formaldehyde (Lillie’s fluid, Sigma-Aldrich, Denmark) for 24 hours at RT. Fixed FDC explants were embedded in paraffin and cut into 5 µm sections for immunostaining. Immunostaining (IHC) was carried out using 200 ng/mL monoclonal antibody, MAb-9B4-Epitope-4, recognizing the N-terminal, extracellular domain end of the CTR (Welcome Receptor, Australia) [41]. After deparaffinization and rehydration, sections were demasked with citrate buffer, pH 6.0, and overnight incubation at 60°C. Unspecific protein binding was blocked with 0.5% casein in TBS buffer for 20 min at RT, followed by washing and the addition of primary antibody, mouse IgG (isotype control, Dako, Denmark) or incubation buffer alone (TBS). Immunoreactivity was detected using the Envision anti-mouse HRP system (Dako, Denmark) with diaminobenzidine (DAB, Dako, Denmark) as the chromogen. Sections were counterstained with Mayer’s acidic hematoxylin for 12sec, rinsed and mounted. Digital histographs were taken using an Olympus BX60 microscope and Olympus C5050-zoom camera.

### Biochemical Markers of Cartilage Turnover

All the biochemical markers were measured in the conditioned medium in which the FDC explants were cultured. Released MMP-derived aggrecan degradation fragments were measured by the AGNxII (aka. 342-G2) assay (Nordic Bioscience, Herlev, Denmark), which is a sandwich ELISA using the monoclonal antibody AF28 specific for the MMP-cleavage site ^342^FFGV, and 2C10 specific for the G1 domain, as a biotinylated catcher and a peroxidase-labeled detector, respectively [39]; [40]. Type II collagen degradation was measured by the competitive ELISA assay C2M (Nordic Bioscience, Herlev, Denmark) that applies the peroxidase-labeled NB44-3C1 monoclonal antibody, specific for the MMP cleavage site RDGAAG^872^ of type II collagen [38]. Both the C2M and the AGNxII followed the procedure described in previous publication by our lab. Type II collagen formation was assessed by the P2NP ELISA (Nordic Bioscience, Denmark), which utilizes a monoclonal antibody NB208-2F8 against the EPIKG internal epitope in the N-terminal type II pro-collagen domain. The specificity of the antibody was tested with nonsense and missense peptides. Sensitivity was tested using samples with high amount of type II collagen propeptides supernatants from IGF-1 stimulated cartilage explants [42], human amniotic fluid (Sunny Lab, Sittingbourne, UK) and fetal calf serum (Sigma-Aldrich) (unpublished). Amniotic fluid was bought from Sunnylab, a UK based company. Sunnylab has retrieved all the necessary inform consent according to demands of the US authorities. The samples were anonymized by Sunnylab before dispatchment. Fetal calf serum was bought from Life technologies. Following statement is given by the manufacture: “We manufacture FBS in compliance with the Food and Drug Administration’s (FDA) Quality System Regulation (cGMP) at our ISO-9001 certified facility in Grand Island, New York. In our U.S. and New Zealand facilities, we process raw FBS under the controls and conditions for the manufacture of medical devices as defined by the FDA. Comprehensive documentation ensures traceability and control of the process.”

Streptavidin-coated micro-titer plates (Roche Diagnostics, Germany) were coated with 1.25 ng/mL biotinylated EPIKG peptide and incubated for 30 min at 20°C before being washed five times in PBST buffer. Meanwhile, a two-fold calibration curve was prepared starting from 2000 ng/mL free EPIKG peptide. 20 µL of conditioned medium and calibrators was added to the plate and then 100 µL peroxidase-labeled NB208-2F8 antibody [20 ng/mL]. After 90 min incubation at 4°C, the plate was washed before 100 ul 3,3′,5,5′-tetramethylbenzidine (TMB substrate, Kem-En-Tec, Denmark) was added and the plate incubated for 15 min at 20°C. The color reactions were stopped with sulfuric acid and read at 450 nm on a standard plate reader. The readings were read from the 4-parameter calibrator curve.

### Measurements of Alkaline Phophatase Activity

Alkaline phosphatase activity was measured in the conditioned medium after ended culturing by a modification of the method of Bessey et al. using p-nitrophenyl phosphate (pNP) as the substrate. 20 µl sample or standard (recombinant alkaline phosphatase) by 80 µl substrate mixture (1% 2-amino-2-methyl-1-propanol, 10.1 mM pNPP, 18 mM MgCl_2_, pH 10) was added to a microtiter plate, and incubated for 20 minutes in the dark at RT. The hydrolysis of pNP to p-nitrophenol (PnP) was stopped by adding 100 µl 0.5 M NaOH to the solution. The color change was monitored at 405 nm on a standard plate reader.

### RNA Purification

Four independent experiments were done with three replicates per treatment. Each replicate represent a pool of five plugs from the same cow. Due to difficulties in obtaining RNA from cartilage tissue, an indirect method of enzymatic digestion was used to isolate chondrocytes for their RNA. Chondrocytes were isolated from the extracellular matrix first by adding 1.4 mg/mL pronase E and leaving the material for 1 hr, and then 1.5 mg/mL collagenase was added and incubated for 5–6 hrs at 37°C. Chondrocytes were harvested by centrifuge at 250 g for 5 min. The purification of total RNA from chondrocytes was carried out using Absolutely RNA Miniprep Kit (Stratagene, US). RNA integrity, quality and quantity were assessed using an Agilent 2100 Bioanalyzer (Agilent Technologies, Santa Clara, CA, USA). The RNA was either used immediately or stored at −80°C.

### Quantitative Polymerase Chain Reaction

200 ng of total RNA from each of the replicates was used for cDNA synthesis using the SuperScript™ First-Strand Synthesis System for RT-PCR kit (Invitrogen, US). Subsequently, the following molecular markers of hypertrophic chondrocytes were measured by real-time quantitative polymerase chain reaction (qPCR): type X collagen (COL10A1), alkaline phosphatase (ALP), Indian hedgehog (IHH), and MMP13. QPCR was also used to measure expression level of CTR. Primer sequences are given in table 1. The qPCR was performed using SYBR Green Supermix (Agilent, US). Reactions were processed with 5 ul of cDNA samples in 25 ul final volume. All PCRs stopped after 40 cycles followed by denaturation at 95°C for 10 min. Each cycle contained annealing for 30 seconds at 56°C for measurement of GAPDH, at 53°C for ALP and MMP13, at 51°C for IHH and COL10A1; extension at 72°C for 30 seconds for all reactions and a final extension at 72°C for 15 min. The relative gene expression level was calculated by the comparative Ct method (i.e. 2^−ΔCt^ method), and expressed as 2∧-([delta]C_t, sample_–[delta]C_t,ref_ ).

**Table 1:** List of primer sequences applied in quantitative PCR analysis.

Gene	Forward	Reverse	Description
COL XNM_000493/ X73320	5′CTGAGCGATACCAAACACCTAC’3	5′GGATGCCTTGCTCTCCTC’3	Marker of hypertrophy
ALPNM_176858	5′TGCGACTGACCCTTCACTCTC’3	5′CACCAGCAGGAAGAAGCCTTT’3	Marker of calcification
IHHNM_001076870	5′ATCTCGGTGATGAACCAGTG’3	5′CCTTCGTAATGCAGCGACT’3	Marker of differentiation and pre-hypertrophy
MMP13NM_174389	5′ACATCCCAAAACGCCAGACAA’3	5′GATGCAGCCGCCAGAAGAAT’3	Marker of chondrocyte activation and differentiation
CTRNM_001076269	5′GGCTGCCAAAGGGTAAC’3	5′AAATCTTGCAGCTCACCG’3	
GAPDHNM_001034034	5′CCTGGAGAAACCTGCCAAGTAT’3	5′AATGAGCTTGACAAAGTGGTCGTT’3	House holding gene

### Measurements of Cyclic Adenosine 3′,5′-monophosphate

CTR down-stream signaling was quantified by cyclic adenosine 3′,5′-monophosphate (cAMP) levels. FDC explants were incubated with or without 20 ng/mL T3 for 0, 4 and 8 days. Then the explants were incubated with 100 µM of the phosphodiesterase inhibitor 3-isobutyl-1-methylxanthine (IBMX) for 2 hours and next treated with either 10 nM salmon calcitonin or 10 nM human calcitonin, or 100 µM forskolin (as a positive control). Explants treated with IBMX alone served as background controls. All compounds were obtained from Sigma-Aldrich, UK. After treatment, the explants were lysed using a 3.5 U/mL papain solution for 24 hours at 60°C. Measurement of cAMP was performed using the HTRF® cAMP cell-based assay (Cisbio Bioassays, US).

### Statistics

Statistical analyses were performed using one-way ANOVA analysis with Dunnett’s post-test or Student’s *t* test. Differences between mean values were considered as statistically significant if p<0.05. N represents number of independent experiments (different cows), whereas n represents number of explants (replicates) in each experiment. Expression data are shown as mean with 95% confidence interval (CI). Biomarker results are shown as geometric mean with 95% confidence interval (CI).

## Results

### Expression of Prehypertrophic Markers and the CTR in T3-induced Cartilage Explants

**Figure 1:**
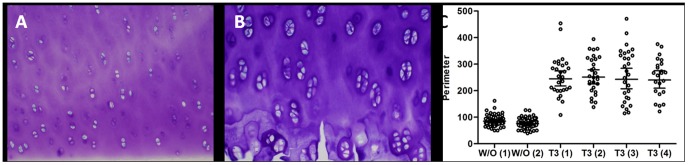
The effect of T3 stimulation on chondrocyte morphology in articular cartilage explants after 4 days of T3 administration. A) Control section (w/o) (n = 2). B) T3-induced explant (n = 4). C) Measurement of the perimeter in cells of the deep zone.

It has previously been shown that T3 induces early chondrocyte hypertrophy [43]. These data was confirmed in a single experiment in current study. T3 administered at 20 ng/mL to cartilage explants over four days did not change the viability of chondrocytes, as measured by Alamar blue (data not shown). T3 induced a prehypertrophic morphological phenotype in the deep zone of the cartilage compared to control (w/o) (fig. 1A, B). No notable changes were observed in the upper zone of the cartilage (data not shown). The size of the lacunas was measured giving the perimeter. It was found that there was a clear separation between w/o and T3-induced explants (fig. 1C).

The mRNA expression of early hypertrophic markers was investigated. Compared with control (w/o, explants not subjected to T3), ALP expression was significantly increased after T3 induction (table 2). MMP13 and IHH were likewise significantly increased in the T3-induced cartilage explants (table 2). COL10A1 expression was also increased, however not significantly (table 2). CALCR expression was significantly increased in T3-induced explants compared to controls (table 2).

**Table 2:** Fold expression (mean(95%CI)) of pre-hypertrophic markers and the CTR in T3-induced explants compared with control (w/o).

	w/o	T3	P	N	n (w/o, T3)
ALP	1.38 (0.75–2.52)	26.2 (5.2–132.2)	0.0011	4	10, 14
IHH	1.53 (0.69–3.46)	4.29 (1.48–12.5)	0.021	3	8, 10
COL10A1	1.44 (0.45–4.55)	3.32 (0.93–11.8)	Ns	3	8, 10
MMP13	1.43 (0.74–2.77)	7.61 (5.03–11.5)	0.0002	4	10, 14
CALCR	1.87 (0.96–3.64)	12.3 (6.93–21.8)	0.0003	2	6, 6

### Immunolocalization of the CTR in T3-induced Explants

**Figure 2:**
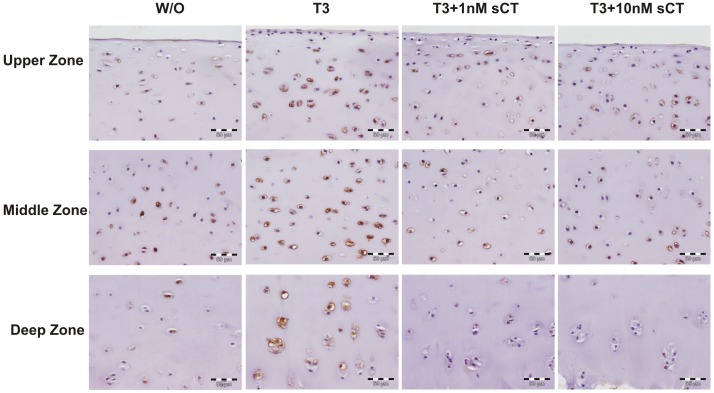
Immunolocalization of the calcitonin receptor (CTR) in cartilage explants induced with or without T3 for 4 days. First panel shows the superficial layer of the cartilage, second panel the mid layer of the cartilage and the third panel the deep layer of the cartilage. Treatment scheme is indicated at the top of the pictures. The pictures are representative of 3 replicates from the same experiment. Positive staining is visualized by brown color.

The membrane expression of the CTR was investigated by immunohistochemistry after four days of T3 and sCT co-stimulation. The CTR was expressed by many cells in the upper and mid cartilage zone, and there seemed to be no notable difference between the w/o and T3-induced explants when looking at the upper and middle zone (fig. 2). In a semi-quantitative assessment it was found that 34–54% of the cells in the upper zone of the control explants and 53%–64% in the T3-induced explants were positive for the CTR (N = 2). The CTR was expressed in the middle zone by 24–57% in the controls and 33–63% in the T3-induced (N = 2). And in the deep zone 45–67% of the cells in the controls and 66–67% of the cells in the T3-induced explants were positive for the CTR (N = 2) (fig. 2). Although immunoreactivity was observed in both w/o and T3-induced explants, there was a tendency toward more intense staining in the T3-induced explants (fig. 2); however this is subjective observation. Co-stimulated with 1 nM and 10 nM sCT reduced the number of positive cells to less than 43% in the deep layer of the explants (fig. 2).

### Cellular and Extracellular Effects of Salmon Calcitonin on T3-induced Explants

It was investigated next whether co-stimulation with T3 and salmon calcitonin (sCT) could change the expression pattern of the chondrocytes as observed by immunohistochemistry. Compared with T3-induced explants, ALP was significantly decreased in response to 1 nM sCT, (table 3). IHH was significantly decreased in response to 1 nM sCT (table 3). There was a tendency towards decreased COL10A expression, but the difference was not significant (table 3). MMP13 expression was significantly decreased in response to 10 nM (table 3).

**Table 3:** Fold expression (mean(95%CI)) of pre-hypertrophic marker in response to 1 and 10 nM sCT stimulation of T3-induced explants compared with T3-induced alone (T3).

	T3	T3+1 nM sCT	T3+10 nM sCT	W/O
ALP (N = 3, n = 9)	38.0 (15.8–91.5)	6.67 (2.96–15.0)	20.6 (5.84–72.3)	1.54 (0.85–2.77)
	0.0001§	<0.05#	ns	<0.001#
IHH (N = 3, n = 9)	3.32 (2.04–5.40)	1.47 (0.62–3.67)	2.64 (1.65–4.22)	1.37 (0.76–2.47)
	0.021§	<0.05#	ns	<0.05#
COL10A1 (N = 3, n = 9)	2.03 (0.91–4.53)	0.58 (0.13–2.63)	2.03 (1.15–3.59)	1.43 (0.76–2.68)
	ns	ns	ns	ns
MMP13 (N = 3, n = 9)	6.26 (4.21–8.31)	3.39 (1.21–9.42)	1.90 (1.04–3.48)	1.42 (0.93–2.16)
	0.0002§	ns	<0.01#	<0.001#

§ P-value of ANOVA test,

# P-value of Dunnett’s post-test.

**Figure 3:**
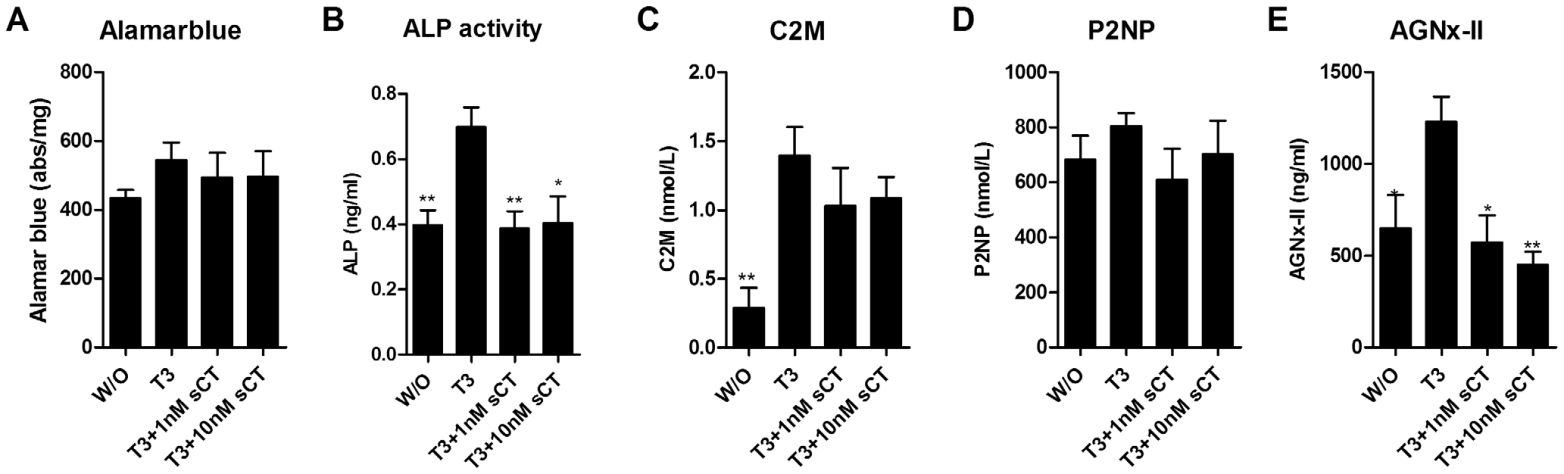
The effect of co-stimulation with salmon calcitonin (sCT) on cartilage turnover. A) Cell viability, measured by Alamar blue, of the cartilage explants. B) Induction of alkaline phosphatase (ALP) activity in the conditioned medium. C) Measurement of matrix metalloproteinase (MMP)-derived type II collagen degradation fragments (C2M) released to the conditioned medium. D) Measurement of type II procollagen (P2NP) released to the conditioned medium. E) Measurement of MMP-derived aggrecan collagen degradation fragments (AGNx-II) released to the conditioned medium. Comparisons were performed by one-way ANOVA with Dunnetts post test. Significance levels; *p<0.05 and **p<0.01, data shown as mean with CI-95% n = 6.

Subsequently, sCT’s anabolic and catabolic effects on cartilage were investigated. Co-stimulation with salmon calcitonin did not change the degree of cell viability measured by Alamar blue in explants treated with T3 alone (fig. 3A). The ALP activity was reduced to the level of the control W/O (fig. 3B). No significant differences in type II collagen turnover measured by C2M and P2NP (fig. 3C and 3D) were observed in the explants treated with salmon calcitonin and the control samples. In contrast, aggrecan degradation was significantly inhibited by both doses of sCT (fig. 3E).

### Induction of Cyclic AMP in T3-induced Explants

**Figure 4:**
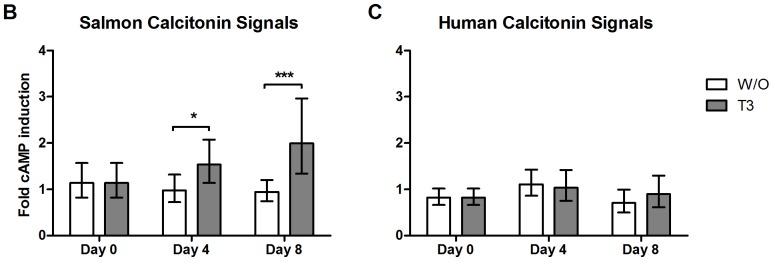
Expression and induction of the calcitonin receptor (CTR) as a result of T3 stimulation. The level of cAMP produced by the chondrocytes two hours after application of A) salmon calcitonin (sCT) or B) human calcitonin (hCT). The measurements were taken at day 0, 4 and 8 in explants with or without T3. The measurements were normalized to vehicle control for each time point, giving the fold induction. Significance level; *p<0.05, and ***p<0.001, data shown as geometric mean with 95%-CI (N = 2, n = 10).

Cartilage explants were preincubated with the nonselective phosphodiesterase inhibitor IBMX to retain the cAMP concentration upon induction of the CTR receptor. Induction of CTR, measured by cAMP, was increased in the T3-induced explants treated with 10 nM sCT compared with the control (fig. 4A). The effect was even higher when the explants were stimulated with T3 for 8 days (fig. 4A). To investigate whether the effect was related to the CTR alone or in conjunction with receptor activity modifying proteins (RAMPs), equal amounts of human calcitonin, which is known not to activate CTR in the presence of RAMPS, were applied to the explants. Human calcitonin [10 nM] did not have the same effect as salmon calcitonin on increasing cAMP release (fig. 4A).

## Discussion

This study investigated the presence and function of the CTR in T3-induced bovine articular cartilage explants. First of all, we confirmed that T3 could induce early hypertrophy of the chondrocytes in the explants: expression of hypertrophic markers was increased and chondrocyte enlargement (pre-hypertrophy) and proliferation was observed, compared with untreated controls. Furthermore, an elevation in MMP activity measured by release of degradation fragments of type II collagen and aggrecan was observed. Interestingly, these T3-induced chondrocytes had an elevated expression of the CTR, which suggests that CTR expression is associated to early chondrocyte hypertrophy. This was further supported by immunolocalization of the receptor to the chondrocytes. Different phenotypes of chondrocytes stained positive for the CTR. There was a tendency toward more intense staining in the T3 induced explants compared to controls. This was most pronounced in the deep layer of the explants were the morphological shift was more evident. However, a more thorough investigation needs to be conducted to quantify the extent of the immune-localization and the difference induced by T3. Notably, T3-induced explants treated with salmon calcitonin seemed to present less CTR, which was expected, since the receptor is internalized as a consequence of sCT binding [44]; [11]. This was further supported by an elevated cAMP release upon treatment with salmon calcitonin from T3-induced cartilage explants compared to controls. Human calcitonin, however, did not have the same effect of raising cAMP levels, which could indicate that the response is dependent on RAMPs (e.g. the amylin receptor). The exact receptor subtype needs to be confirmed in more detail in another experimental set-up. Moreover, when co-stimulating the explants with the most potent concentrations of salmon calcitonin [24], the cartilage appeared to be protected from hypertrophy. Compared with explants treated with T3 alone, the expression of the hypertrophic markers was reduced in the co-stimulated explants and aggrecan degradation was prevented. In summary, we showed that T3-induced chondrocytes expressed the CTR and that induction of the receptor seemed to protect the cartilage from hypertrophy. These are the first data to show a direct connection between early chondrocyte hypertrophy in cartilage explants and the potentially protective effect of the CTR.

The effects of calcitonin on bone have been demonstrated previously to be mediated by direct binding of the hormone to CTRs expressed in osteoclasts in their basolateral membrane [45]. In contrast, no conclusive evidence on the expression of the CTR in cartilage has been provided. Our group previously reported the existence of an intron spanning sequence of the coding region of CTR mRNA, as well as the full-length transcript, in human articular cartilage [13]; [19]. Furthermore, we showed that the receptor on the chondrocytes’ surface was a functional receptor [14]. In contrast, Lin and colleagues concluded that the CTR was not expressed in human cartilage after investigating its existence in tissue and isolated chondrocytes by PCR, western blotting and immunochemical analysis [15]. Nevertheless, the CTR was identified and found to be functional in osteocytes [46], adding to the growing evidence that CTR is expressed in cells of the mesenchymal lineage as chondrocytes.

Thus, there has been some debate as to whether the CTR is expressed by chondrocytes [46]. An important contributor to this debate has been the use of different *in vitro* systems. There is evidence that culturing chondrocytes as an adherent monolayer invariably leads to a process of differentiation whereby cells adopt a fibroblastic morphology, lose their chondrocyte-specific gene-expression pattern and initiate or upregulate the expression of fibroblast-associated genes such as type I, III and V collagens and versican [47]; [48]; [49]. Therefore, chondrocytes differentiated on plastic with fetal bovine serum cannot be used as a representative sample of cartilage cell biology, particularly in regards to cartilage gene expression profile. It is more meaningful to study chondrocytes in their native micro-environment – the cartilage matrix. The cartilage matrix is a compact, collagenous and hypoxic entity, which depends on diffusion for nutrients. The cartilage seems to be rather protective of the chondrocytes, protecting them from differentiation and exogenous factors [50]. Joint degenerative diseases are partly characterized by loss of structural integrity and degradation of the cartilage, leading to the loosening and exposure of the chondrocyte to its surroundings. This results in the initiation of a pathway of cellular differentiation and hypertrophy [1]. Targeting cartilage for drug development should include investigation of the relevant chondrocyte phenotypes. The explant model presented here might be a good *in vitro* model for investigating the mode of action of candidate drugs on articular cartilage.

The present study clearly demonstrates that the CTR is more highly expressed in hypertrophic than normal chondrocytes, and that early hypertrophic chondrocytes respond to salmon calcitonin. Interestingly, human calcitonin did not affect the chondrocytes. Because human calcitonin only activates the CTR in the absence of RAMPs [51], our findings suggest that RAMPs must have been present, together with the CTR, in the explants respongding to salmon calcitonin.

In conclusion, the investigation of pharmacological interventions for OA should focus on the correct phenotype of chondrocytes. In the present study we clearly demonstrated that hypertrophic chondrocytes have a different phenotype than normal chondrocytes, they express more of the CTR, and they respond to salmon calcitonin.
